# Colonization of *Anopheles cracens*: a malaria vector of emerging importance

**DOI:** 10.1186/1756-3305-6-81

**Published:** 2013-03-28

**Authors:** Amirah Amir, Jia Siang Sum, Yee Ling Lau, Indra Vythilingam, Mun Yik Fong

**Affiliations:** 1 Department of Parasitology, Faculty of Medicine, University Malaya, Kuala Lumpur, 50603, Malaysia

**Keywords:** Vector, Transmission, Malaria, Gonotrophic cycle, Lifecycle, *Anopheles cracens*

## Abstract

**Background:**

*Anopheles cracens* has been incriminated as a vector for the simian malaria parasite, *Plasmodium knowlesi,* that is the fifth *Plasmodium* species infecting humans. Little experimental data exists on this mosquito species due to the lack of its availability in laboratories.

**Findings:**

The population of *An. cracens*, collected from Kuala Lipis, Pahang was maintained at the insectary of the Department of Parasitology, Faculty of Medicine, University Malaya at 24-26°C and 60-80% relative humidity. The mosquitoes were maintained with artificial mating and blood-fed on humans and hamsters. The colony has been established since November 2011 and to date has reached its sixth generation.

**Conclusion:**

This is the first description of maintaining the Malaysian strain *An. cracens* colony by artificial mating. Colonization of *An. cracens* will provide fundamental information for genetic studies and will be useful in assessing comparative susceptibility to *Plasmodium* parasites.

## Findings

### Introduction

The *Anopheles Leucosphyrus* group of mosquitoes play a significant role as simian malaria vectors in South-east Asia. Three of its members which are known to be efficient vectors for human malaria parasites include *An. balabacensis* Baisas, *An*. *dirus* Peyton and Harrison, and *An. leucosphyrus* Doesnitz (now known as *An. latens*) [1]. Species of the *An. dirus* complex can be found from India to Taiwan and from the 30° north parallel to the Malaysian peninsular and the northern tip of Sumatra, Indonesia [2]. *Anopheles cracens (=An. dirus* B) [3] was found in southern Thailand, Perlis, Terengganu (peninsular Malaysia) and Sumatra, Indonesia [3,4]. Recent studies have shown that *An. cracens* is also present in Kuala Lipis Pahang (peninsular Malaysia) [5,6].

A study comparing seven South-east Asian *Anopheles* species with *An. dirus* showed that *An. cracens* has one of the highest susceptibilities to *Plasmodium cynomolgi* B strain (simian malaria) [7]. Besides being recently established as the main vector for *P. knowlesi* in Kuala Lipis*, An. cracens* has also been proven to be an efficient laboratory vector for both *P. falciparum* and *P. vivax*[5,8]. Upon comparing filarial vector competence between *An. stephensi*, *Aedes aegypti*, *An. gambiae* and *An. cracens,* the latter was shown to be involved in the transmission of *Brugia pahangi*[9].

Many aspects of the vector-parasite relationship need to be studied to better understand their importance in the epidemiology of knowlesi malaria. These studies await the availability of an adequate supply of laboratory bred colony material. Thus, the current study presents the successful colonization and maintenance of *An. cracens* in the laboratory.

## Methods

A total of 41 female *An. cracens* were caught using the bare leg landing method in Kuala Lipis, Pahang (N04°12.584’ E101°52.515’) in November 2011. This project was approved by the Ethical and Research Review Committee of the Ministry of Health, Malaysia NMRR-11-1050-110619. Two of the caught *An. cracens* were genotyped, two more were pinned as a reference collection and the remaining 37 female mosquitoes were used for establishment of the colony which to date has reached its sixth generation. The collection was carried out between 18:30 and 21:30 hours for two consecutive days. Each mosquito was caught using a 50 × 19 mm specimen glass tubes with its base covered in moist tissue paper to provide humidity and its top covered with cotton wool to prevent escape. The mosquitoes were morphologically identified using keys of Reid and Sallum [3,10].

DNA from two morphologically identified *An. cracens* were extracted for rDNA *ITS2* and cytochrome oxidase c subunit I (COI mtDNA) sequence analysis [11-13]. The rDNA *ITS2* was amplified using primers ITS2A and ITS2B. PCR was performed according to Beebe and Saul [11]. The COI gene was amplified using primers UEA9.2 and UEA10.2. PCR was performed according to Sallum *et al.*[13]. The PCR products were sent to a commercial laboratory for sequencing.

The remaining caught *An. cracens* were transferred into paper cups covered with netting lids and blood fed by introducing a human arm. After two days, five blood fed mosquitoes were transferred to each oviposition pot (9 cm in diameter, 7 cm high) lined with wet filter paper and covered with a netting lid. Eggs laid by these mosquitoes were used to establish the laboratory colony.

Upon hatching, the larvae and remaining eggs were transferred into a larval rearing pan (white plastic tray, 20 × 30 × 5 cm), half filled with dechlorinated water. Approximately 200 larvae were transferred into each of these larval rearing pans. The larval food comprised of the following, which were finely ground: 100 g dog biscuits, 200 g nestum, 10 g yeast, 50 g liver powder and 10 g vitamin B complex. To first instar larvae, 0.03 mg larval food was provided and this was gradually increased from 0.03 mg to a maximum of 0.12 mg as the larvae increased in size. Pupae were removed daily with a pipette and placed in plastic containers (9 cm in diameter, 7 cm high) containing dechlorinated water and placed in a screened cage (30 × 30 × 30 cm) for emergence. Emerged adults were provided with a 10% sugar solution with vitamin B complex.

Adult females that were at least five days old were starved for 24 h before being allowed to feed on hamsters or human arm. Engorged females were removed and mated with three to four day old males using the forced mating method as described [14]. Similar to the artificial mating of *An. labranchiae* and *An. freeborni*, removal of the male’s head was not necessary although stimulation of the male was more rapid when decapitated [15,16]. During forced mating, the median time for the mosquitoes to remain joined was 21 s (range: 8–480 s, n = 237) after which, the female is released by the male. The same male was used to mate with a maximum of three females. This was based on Baker’s findings, which showed that insemination occurred only in the first three females [17]. Furthermore, an experiment with one male *An. pseudopunctipennis* mating with three successive females showed that the first, second and third mating led to 70%, 90% and 40% of fertilized females respectively [17,18].

After artificial mating, females were introduced singly into a plastic cup (4 cm in diameter, 5.5 cm high) lined with filter paper and provided with a 10% sugar solution with vitamin B complex. After three days, water was added to the filter paper and female mosquitoes were allowed to oviposit. Up to 60-91% of the females, which laid eggs were from the first mating, followed by 9-40% from the second mating and 7-10% from the third mating. Female mosquitoes that did not lay eggs by day seven and those which had already laid eggs were given a second blood feed before allowing them to oviposit again. The insectory was maintained at 24-26°C at 60-80% relative humidity, illuminated with a combination of natural light and fluorescent lighting for an average of 12 h a day.

## Results and discussion

Sequence analysis of rDNA *ITS2* and cytochrome oxidase *c* subunit I (COI mtDNA) from two morphologically identified *An. cracens* confirmed its species [11-13]. Most comprehensive data was obtained from F2 generation onwards. A total of 517 *An. cracens* made up the F2 generation with a female to male ratio of 1.23:1. This was followed with a total of 519, 272, 182 and 516 *An. cracens,* which made up the F3, F4, F5 and F6 generation respectively. Female to male ratios for F3 up to F6 generation did not vary much, ranging between 1:0.8 to 1:1.06. The maximum lifespan of the adult female and male in our laboratory was 77 and 51 days respectively. A mean of 3.26 males and 3.22 females died each day. The survival rate, defined as the percentage of mosquitoes that survived 30 days, were 13.9% for males and 31.6% for females.

Less than 25% of the adult females which underwent forced mating oviposited, with 18.5% of oviposition occurring by day four post bloodmeal. The remaining adult females oviposited after day five with the longest viable eggs being laid fourteen days after blood feeding. The average number of deposited eggs per individual F2 female was 123.1 ± 71.3 (range: 25–245, n = 9). This figure varied with subsequent generations as shown in Table 1. These numbers are comparable with other laboratory reared *Anopheles* species such as *An. maculatus*, 80–100 eggs per female, *An. albimanus*, 80*–*122 eggs per female and *An. fluviatilis*, 68–78 eggs per female [12,19,20].

**Table 1:** **Laboratory colonization of *An. cracens *under insectory and ambient conditions**

**Generation**	**Percentage of adults (%)**		**Mean no. of eggs laid per female**	**Developmental time from larva to pupa (days)**	**Time of oviposition after blood-feeding (days)**
	**Female**	**Male**			
F2	55.3	44.7	123.1 ± 71.3	7 - 17	4 - 8
F3	48.6	51.4	46 ± 23.7	7 - 24	5 - 13
F4	51.5	48.5	95 ± 43.2	7 - 22	5 - 11
F5	55.5	44.5	90.3 ± 59.6	9 – 25	4–13
F6	49.2	50.8	91 ± 50.3	8 - 19	3 - 18

The eggs hatched after two days, into first instar larvae. Pupation started on the seventh day of hatching. The adults emerged after two days of pupal stage. The observation showed that 62.6%, 77.8%, 65.4% and 87.3% of the eggs laid by F2, F3, F4 and F5 females respectively, successfully matured and emerged into adults.

Blood feeding proves to be challenging in *An. cracens* colonies. Female *An. cracens* did not feed on white mice or gerbils in our laboratory. Hamsters showed potential as some females fed on them. The mosquitoes remain highly attracted to humans for blood feeding. Other *Anopheles* species, which were maintained using hamsters for blood feeding includes *An. philippinensis* and *An. albimanus*[19,21]. Other animals successfully used for blood feeding include rabbits for *An. fluviatilis, An. pseudopunctipennis*[18,20,22] and *An. gambiae* and guinea pig for *An. maculatus*[14].

Although it was found that *An. cracens* (*An. balabacensis,* Perlis form) was a stenogamic species in the laboratory [23], it was not the case with this species in Malaysia. One of the most important requirements for successful colonization is personal dedication and care. This includes carrying out procedures at stipulated times. For example, after blood feeding and mating, mosquitoes must be set for egg laying after 3 days. Larvae should not be over fed. Overcrowding of both larvae and adults should be avoided.

Colonies of free mating *An. cracens* have been established in Chiang Mai University, Thailand [24-26]. However, the rearing protocol was not published. This is the first description of maintaining the Malaysian strain *An. cracens* colony by artificial mating. Gonotrophic cycle was established as 3–5 days. Colonization of *An. cracens* will enable us to gain insight into the evolutionary and speciation history of *An. cracens* specifically and on the *Anopheles* genus as a whole. If possible, we will also be looking at morphological variance with other existing colonies. This colony will also be useful in assessing comparative susceptibility to various *Plasmodium* parasites.

## Abbreviations

rDNA: Ribosomal deoxyribonucleic acid; ITS2: Internal transcribed spacer 2.

## Competing interests

The authors declare that they have no competing interests.

## Authors’ contributions

LYL, FMY and IV conceived the concept of the project. AA, SJS, IV and LYL conducted the field trip and mosquito catching. AA and SJS maintained the mosquito colony in the laboratory. AA wrote the first draft of the manuscript and LYL, FMY and IV revised it. All other authors read and approved the final version of the manuscript.
